# Virus-based pharmaceutical production in plants: an opportunity to reduce health problems in Africa

**DOI:** 10.1186/s12985-019-1263-0

**Published:** 2019-12-30

**Authors:** Pingdwende Kader Aziz Bamogo, Christophe Brugidou, Drissa Sérémé, Fidèle Tiendrébéogo, Florencia Wendkuuni Djigma, Jacques Simpore, Séverine Lacombe

**Affiliations:** 1 Interactions Plantes Microorganismes et Environnement (IPME), IRD, CIRAD, Université Montpellier, 911 Avenue Agropolis BP64501, 34394 Montpellier Cedex 5, France; 2 Laboratoire de Virologie et de Biotechnologies Végétales, Institut de L’Environnement et de Recherches Agricoles (INERA)/LMI Patho-Bios, 01BP476, Ouagadougou 01, Burkina Faso; 3 Laboratoire de Biologie Moléculaire et de Génétique (LABIOGENE), Ecole Doctorale Sciences et Technologie, Université Joseph Ki-Zerbo; Centre de Recherche Biomoléculaire Piétro Annigoni (CERBA), Ouagadougou 01, BP 364 Burkina Faso

**Keywords:** Plant viral expression vectors, Plant-made therapeutics, Recombinant proteins, Neglected diseases, Developing African countries

## Abstract

**Background:**

Developing African countries face health problems that they struggle to solve. The major causes of this situation are high therapeutic and logistical costs. Plant-made therapeutics are easy to produce due to the lack of the safety considerations associated with traditional fermenter-based expression platforms, such as mammalian cells. Plant biosystems are easy to scale up and inexpensive, and they do not require refrigeration or a sophisticated medical infrastructure. These advantages provide an opportunity for plant-made pharmaceuticals to counteract diseases for which medicines were previously inaccessible to people in countries with few resources.

**Main body:**

The techniques needed for plant-based therapeutic production are currently available. Viral expression vectors based on plant viruses have greatly enhanced plant-made therapeutic production and have been exploited to produce a variety of proteins of industrial, pharmaceutical and agribusiness interest. Some neglected tropical diseases occurring exclusively in the developing world have found solutions through plant bioreactor technology. Plant viral expression vectors have been reported in the production of therapeutics against these diseases occurring exclusively in the third world, and some virus-derived antigens produced in plants exhibit appropriate antigenicity and immunogenicity. However, all advances in the use of plants as bioreactors have been made by companies in Europe and America. The developing world is still far from acquiring this technology, although plant viral expression vectors may provide crucial help to overcome neglected diseases.

**Conclusion:**

Today, interest in these tools is rising, and viral amplicons made in and for Africa are in progress. This review describes the biotechnological advances in the field of plant bioreactors, highlights factors restricting access to this technology by those who need it most and proposes a solution to overcome these limitations.

## Background

Least developed countries (LDCs) encounter major health problems that are difficult to solve mainly for economic reasons, such as medicine acquisition and logistic organization. Among the deadly diseases occurring in Africa, human immunodeficiency virus (HIV) disease and malaria are old diseases that still occur with high mortality among the infected population. In 2016, there were 37 million people living with HIV, 1 million HIV deaths and 1.8 million new infections worldwide. Approximately 70% of HIV-infected people live in Africa [1]. In addition, HIV and tuberculosis are often associated diseases in developing countries, and both these diseases reciprocally promote illness and threats to life [2].

Neglected tropical diseases (NTDs) are mainly curable. However, they still cause damage in tropical areas. NTDs impair the lives of 1 billion people worldwide and threaten the health of millions more [3]. While LDCs struggle to solve their NTD-related health problems, new emerging disease outbreaks such as Ebola virus diseases (EVD) have also appeared in recent decades, affecting mainly remote, low-income and politically marginalized populations. EVD has a lethality rate that can reach 90% [4, 5]. In addition to EVD, other severe diseases have occurred in Central and West Africa, with high lethality rates aggravating the health situation in these zones. Dengue fever, zika fever and chikungunya fever are recent viral diseases that target poor populations worldwide. These diseases develop rapidly and spread quickly all over the world, constituting major threats toward global health.

One of the main obstacles to health problem resolution for LDCs is a lack of economic resources. For example, in Burkina Faso, where more than half of the population lives on less than 2 US$ per day, an efficient malaria cure costs approximately 14 US$. Malaria in these areas represents a public health problem with substantial risks for the pregnant woman, her fetus and the newborn child [6]. Vaccination against hepatitis consists of three independent doses costing 15 US$ each. This is not affordable for most of the population, and the governments of these LDCs do not have enough financial means to support these cures.

To manage human diseases such as HIV, malaria or new and emerging diseases, vaccines and therapeutics are crucial. These therapeutics and vaccines need to be inexpensive to be suitable for LDCs. To meet these challenges, plant-produced vaccines and therapeutic agents offer enormous potential for providing relief to LDC healthcare systems. In recent decades, plants have been recognized as a possible solution for global health. Plant-made biopharmaceuticals have long been considered a promising technology for providing inexpensive and efficient medicines for LDCs [7–10]. Indeed, plants meet all the criteria for profitable therapeutic and vaccine production, such as efficiency, reduced cost, temperature stability (can be stored at ambient temperatures for prolonged periods of time) and easy transport, even to remote areas [11–13]. In addition, plant-based production can be scaled efficiently and simply by sowing more seeds [2]. Recently, progress has been reported in oral delivery using lyophilized plant tissues to produce and deliver biopharmaceutical proteins [9, 14]. As a result, plant-produced biopharmaceuticals have the potential to be more accessible to the rural poor.

Plant virus expression vectors have revolutionized the use of plants as bioreactors. Once introduced to host plant cells, viruses engineered to contain a gene of interest replicate, allowing the expression of foreign proteins at high levels in infected plants [15, 16]. Plant viruses have been engineered to either express subunit vaccines or act as epitope presentation systems. Many plant viruses have been used for these purposes. Among them, the most popular vectors are derived from *Tobacco mosaic virus* (TMV), *Potato virus X* (PVX) and *Cowpea mosaic virus* (CPMV) [16, 17]. More recently, plant viruses have been utilized as nanoparticles or virus-like particles (VLP) to transport drugs and active molecules into cancer cells, thus offering a new and potent arsenal for the fight against cancer [18], or used as scaffolds for peptide/epitope presentation as vaccines [13].

**Fig. 1:**
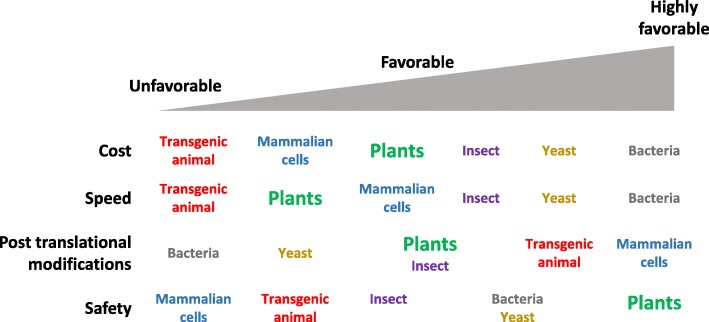
Comparison of the efficiency of protein production systems. Cost, speed, posttranslational modifications and safety are considered (adapted from [19]). Plant production systems noted in green are compared to transgenic animal (noted in red), mammalian cell (noted in blue), insect (noted in purple), yeast (noted in orange) and bacterial (noted in gray) production systems

The driving forces behind the rapid growth of plant bioreactors include low production costs, product safety, posttranslational modifications and easy scale-up compared to other production systems based on bacteria, yeast or mammalian cells [17] (Fig. 1). These attributes have provided plant-made pharmaceuticals with an opportunity to assist people in resource-poor countries to receive medicines that were previously inaccessible. This review describes the current progress and limitations of plant-produced biopharmaceuticals with a particular emphasis on those that target developing countries.

## Main text

### Need for novel platforms for therapeutic protein production for LDCs

Approximately 15 million deaths per year worldwide (> 25%) are estimated to be related directly to infectious diseases [20]. Vaccination is considered the most efficient method of health intervention to combat infectious diseases. However, the high cost of vaccination makes it unaffordable for most people living in developing countries, where the daily average income of nearly one billion people is less than 1 US$ [17]. A 14-fold increase in the cost of vaccines over the past decades has been recorded [21]. Current platforms of therapeutic production, such as bacteria, yeast or mammalian cells, lead to high-cost products. Fermentation, purification, adjuvant association, cold storage, transportation and sterile delivery are some of the expensive aspects of these therapeutics from production to usage. Furthermore, the final products from these expression systems are either not similar to the expected product because of the absence of posttranslational modifications (bacteria) or are similar but not safe because they have been produced in primitive hosts or in hosts closely related to humans that can carry pathogens. For all these reasons, it is necessary to investigate alternative strategies for therapeutic production and delivery with an emphasis on cost, efficiency and safety.

In the early 1990s, plants were recognized as useful bioreactors for the production of high-value proteins [22]. First, plant-derived pharmaceuticals were generated from transgenic plants [11, 22–26]. This was a great opening for complex protein production requiring posttranslational modifications previously unachievable with the traditional fermenter-based expression platforms [27]. However, this technological approach met four main hurdles: time consumption, low level of protein expression, high labor requirements and uncertainty regarding the biological activity of the end product. Moreover, biosafety concerns around the environment containment of transgenic plants and transgene flow had to be taken into account [27–31]. Consequently, there has been an increasing trend toward the improvement of the use of plants as bioreactors in recent years.

The first crucial advance was the use of transient expression systems relying on *Agrobacterium tumefaciens* as a vector to deliver DNA encoding proteins of interest directly into leaf cells by syringe infiltration: agroinfiltration [32]. These transient systems allow the production of ectopic proteins in days rather than the months necessary for stable transgenic expression. Moreover, rapid testing of different genetic constructs could be performed without waiting for plants to be stably transformed, selected and grown. However, the product yield remained relatively limited, constituting a major constraint (Table 1). One of the explanations for this problem is the RNA silencing initiated by plant cells in response to the introduction of foreign nucleic acids, which acts to specifically degrade foreign RNA [42]. RNA silencing is a highly conserved mechanism in eukaryotes with fundamental implications in many biological processes, including defense against foreign nucleic acids such as transgenes or viruses [42, 43]. This hurdle was overcome by the exploitation of viral proteins that have acquired RNA silencing suppression functions during their coevolution with plants [44]. Protein production can be increased by the coexpression of viral proteins that suppress RNA silencing activity. Indeed, the presence of such viral proteins in these transient expression systems can overcome the RNA silencing machinery, avoiding foreign RNA degradation. The inhibitory effect of the P19 suppressor from *Tomato bushy stunt virus* (TBSV) or *Artichoke mottle crinkle virus* (AMCV) on the RNA silencing pathway has been exploited with success to enhance the transient or constitutive expression levels of recombinant proteins in plants [45, 46]. Finally, cocktails of suppressors acting at distinct steps of the RNA silencing pathway have been used to optimize recombinant protein production [40].

**Table 1:** Evolution over time of plant expression system yields. The table contains some selected examples to illustrate the transition between transgenic and transient expression systems from 1999

Plant host	Recombinant protein	Type of Expression system	Highest expression level	Year and Reference
Carrot	GAD65	Transgenic	0.01% TSP	1999 [33]
Potato	NVCP	Transgenic	0.16 mg/g fruit weight	2006 [34]
Tomato	NVCP	Transgenic	0.12 mg/g tuber weight	2006 [34]
*N. benthamiana/ N. tabacum*	2G12	Transient (binary vector)	0.1 mg/g LFW	2013 [35]
*N. benthamiana*	NVCP	Transient (binary vector)	1 mg/g FLW	2013 [36]
*N. benthamiana*	Pfs25	Transient (Viral vector)	0.14 mg/g FLW	2015 [37]
*N. benthamiana*	HPV16 L1	Transient (Viral vector)	0.25 mg/g FLW	2016 [38]
*N. benthamiana*	M2eHBc	Transient (Viral vector)	5–10% TSP FLW	2017 [39]
*N. benthamiana*	PSA	Transient (binary vector)	0.4 mg/g FLW	2018 [40]
*N. benthamiana*	HPV 16 L2 peptides (SAC 108–120)	Transient (Viral vector)	0.145 mg/g FLW	2019 [41]

*GAD65* human glutamic acid decarboxylase, *NVCP* Norwalk virus capsid protein, *2G12* anti-HIV neutralizing monoclonal antibodies, *Pfs25* Pfs25 protein expressed on the surface of *Plasmodium falciparum* gametes, zygotes and ookinetes, *M2eHBc* influenza virus M2 protein (M2e) fused to hepatitis B core antigen, *PSA* promastigote surface antigen of *Leishmania infantum, HPV16 L1, L2* major capsid protein of *HPV 16, TSP* total soluble protein, *LFW* leaf fresh weight

**Fig. 2:**
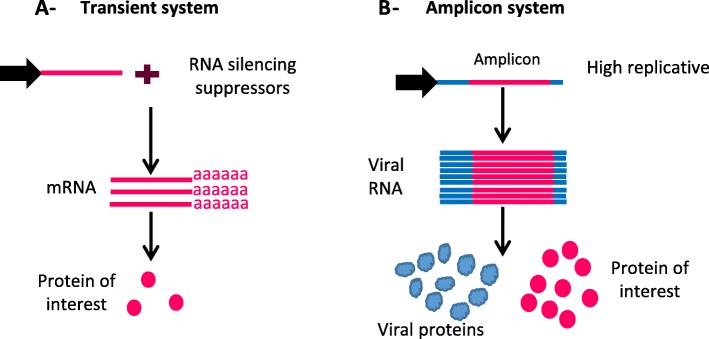
Schematic representation of gene of interest overexpression through **a-** transient expression system using viral RNA silencing suppressor and **b-** engineered amplicon vectors based on viruses. Genes encoding proteins of interest are represented as pink lines and the viral genome is represented as blue lines. Promoters driving constructs are represented as black arrows

Another significant development in the plant bioreactor field was the coupling of agroinfiltration with the delivery of cDNA encoding viral RNA, which is used as an “amplifier” for production of the protein of interest. Once introduced into host plant cells, viruses engineered to contain a gene encoding a candidate protein replicate many times, and the corresponding protein can be produced in higher quantities than in a nonviral context [16, 47] (Fig. 2).

Transient expression systems exploiting either RNA silencing suppression or viral-based vectors have revolutionized the use of plants as bioreactors. They shorten production time and increase the yield of the protein of interest while avoiding the concerns of the general public about the use of stably genetically modified organisms. The use of plant virus suppressors of RNA silencing was demonstrated to increase the expression levels of heterologous proteins several-fold in agroinfiltrated leaves [42]. Virus vectors allow the expression of foreign genes at higher levels in infected tissues than in stably transformed plants [48]. All these advances make plant bioreactor expression systems convenient to address health problems in developing countries.

### Plant viral vectors: state of the art and production systems

The first discovery of a virus in the nineteenth century has led to the emergence of virology and important biotechnological advances. In the field of biotechnology, plant viruses have become the basis of practical tools for plant fundamental genomic studies and protein production. The first exploited virus, TMV (*Tobacco mosaic virus*), has been established as a viral vector and enabled the production of diverse proteins of interest [45, 47–49]. From this first advance to the present, scientists and industry have continued to improve viral vectors to develop inexpensive and efficient expression systems for the production of recombinant proteins. The science of plant vectorology has evolved substantially from this first finding. Plant virus expression vectors have been designed from genomes of both positive-sense RNA viruses and single-stranded DNA viruses (Table 2) [10, 16, 47, 62].

**Table 2:** Description of some of the main viral expression vectors currently used for heterologous protein expression

Virus	Family	Shape /Type of genetic material	Usage	Selected references /examples
PVX	*Potexviridae*	Filamentous virus (+) ssRNA	Expression vector Nanoparticle (VLPs)	[50, 51]
PapMV (*Papaya mosaic potexvirus*)	*Potexviridae*	Filamentous virus (+) ssRNA	Expression vector Nanoparticle (VLPs)	[52, 53]
TMV	*Tobamoviridae*	rod-shaped virus (+) ssRNA	Expression vector Nanoparticle (VLPs)	[54, 55]
SHMV (*Sun hemp mosaic virus*)	*Tobamoviridae*	rod-shaped virus (+) ssRNA	Expression vector	[56]
CPMV	*Comoviridae*	Icosahedral virus (+) ssRNA	Expression vector Nanoparticle (VLPs)	[57]
BeYDV	*Geminiviridae*	Icosahedral virus (+) ssDNA	Expression vector	[58, 59]
BCTV (*Beet curly top virus*)	*Geminiviridae*	Icosahedral virus (+) ssDNA	Expression vector	[60]
TYDV (*Tobacco yellow dwarf virus*)	*Geminiviridae*	Icosahedral virus (+) ssDNA	Expression vector	[61]

*ssRNA* single strand RNA, *ssDNA* single strand DNA, *VLPs* Virus like particles

**Fig. 3:**
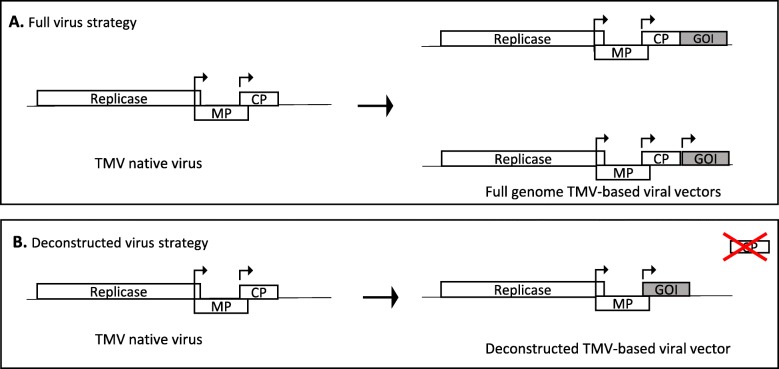
Simplified diagram of a plant viral based expression vector. TMV virus has been taken here as a model virus for viral vector construction illustrations. The figure have been adapted from Liu et al. [56]. **a-** Viral expression vector constructions based on the full virus strategy, upper part: the gene of interest is under the control of TMV CP promoter. Lower part: the gene of interest expression is governed by a new promoter. **b-** Vector construction based on the deconstructed virus strategy. ORFs are labeled in each box. MP: movement protein, CP: coat protein, GOI: gene of interest, arrow: promoter

Plant viral vectors can be divided into three categories based on the manner they have been Plant viral vectors can be divided into three categories based on the manner in which they were designed. The first strategy uses the unmodified native virus to maintain its full properties. It is called the “full-virus strategy”. Therefore, this first strategy led to the design of fully functional viruses that, despite carrying and expressing heterologous sequences, have retained infectivity, stability and systemic virulence in their hosts. The foreign gene of interest is expressed either as part of a fusion protein with a viral protein or separately from an additional strong subgenomic promoter that is incorporated into the viral genome (Fig. 3**.**a). Several scientific works have reported the successful expression of different immunogenic epitopes as fusions with viral coat proteins [62, 63]. In some cases, these epitopes were localized to the surfaces of the virus particles [63]. Furthermore, immunogenicity has been demonstrated in rodent models for some of them [64]. This kind of vector theoretically allows the expression of useful quantities of the protein of interest, approximately 10% of total soluble protein, similar to the amount of coat protein produced by a wild-type virus. This amount is considered a biological limit [65]. However, the full-virus strategy presents several limitations, such as restrictions with respect to the size of the foreign protein that can be expressed [66]. There are potentially some problems with the stability of the genome, which can result in total or partial excision of the foreign genes. The other limitation is safety, as the amplicon is autonomous and can potentially spread in the environment. Such autonomous viral constructs are not suitable for developing countries because of biosafety concerns. To produce proteins in safer conditions, scientists have moved to another type of viral vector technology called the “deconstructed virus strategy” (Fig. 3.b). Deconstructed vectors are composed solely of the genomic regions required for viral replication, called the viral amplicons [67]. The deconstructed virus strategy aims to replace an ORF of the native virus that is not necessary for virus replication with the foreign gene of interest. This would allow incorporation of a gene of interest of a larger size than in the case of the “full-virus technology”. Among the functions necessary for a virus to perform a successful infection (initial host infection, nucleic acid replication, protein translation, assembly of mature virions, cell-to-cell spread, long-distance spread, reprogramming of the host biosynthetic processes, suppression of silencing, etc.), not all of these functions are necessarily required for the viral vector expected to be expressed in a transitory and local manner within a tissue. On the other hand, other functions, such as high-level expression, are essential. The missing protein encoding the unnecessary ORF that has been deleted and replaced by the gene of interest can be provided *in trans* to participate in chimeric virus multiplication. This *trans* complementation could be achieved through coagroinfiltration [68] or establishment of transgenic plants expressing the missing protein [69–73]. Thus, for example, the agrodelivery of the two genomic components of CPMV by a mixed suspension of bacterial cultures, each harboring different subgenomic complements, can lead to a high level of expression of the protein of interest, such as the Green Fluorescent Protein (GFP). This result confirmed the utility of this *trans*-complementation approach for the accurate use of CPMV-based vectors for protein expression [68]. Open reading frame 3a (ORF3a) frameshift and deletion mutants of *Cucumber mosaic virus* (CMV) accumulate to the wild-type level only when inoculated in transgenic tobacco expressing the removed open reading frame [72]. Here, in the case of the deconstructed virus strategy, the use of the amplicon is secure because it is not autonomous. This category of constructs is suitable for developing countries because it is more manageable than the former; its utilization can be safely achieved in containment greenhouses adapted for transgenic cultures (level S2).

More recently, plant viruses have been utilized as nanoparticles to transport drugs and active molecules into cancer cells. Plant viruses play a key role in the fight against cancer by acting as shuttles that are able to reach tumor cells and deliver drugs or elicit a highly localized and powerful immune response [74]. This third category of plant viral vectors has been established for viruses where X-ray crystallography structures were available. Several viruses have been examined as drug delivery vehicles (Table 3). A *Red clover necrotic mosaic virus* (RCNMV) encapsulating the anticancer drug doxorubicin (DOX) worked more efficaciously than pegylated liposomal doxorubicin (PLD) in an ovarian cancer model [81]. Moreover, the RCNMV drug carrier was delivered more efficiently to tumors than PLDs and was cleared more rapidly from the plasma [81]. Another example has been produced with *Hibiscus chlorotic ringspot virus* (HCRSV). The authors demonstrated its ability to transport the drug molecules polystyrene sulfonic acid (PSA) and polyacrylic acid (PAA) acting on cancer cells [77]. They also demonstrated that HCRSV VLPs conjugated with folic acid or encapsulating doxorubicin represent a delivery system that could elicit cytotoxicity in human ovarian cancer cells [82].

**Table 3:** Selected examples of plant virus nanoparticles tested on some cancers

Plant Virus	Application Tested	Carried Drug	Reference
CPMV	Solid tumors	Ag	[75, 76]
HCRSV (*Hibiscus chlorotic ringspot virus*)	Ovarian cancer	DOX	[77]
TMV	Ovarian cancer	cisplatin	[78]
PapMV	Murine B melanomas	immune checkpoint blockade, immunomodulation or therapeutic vaccination	[79, 80]

*DOX* doxorubicin

These three strategies, the full-virus, deconstructed virus and nanoparticle strategies, have been exploited for the construction of several viral expression vectors based on viruses belonging to some families, including Potexviruses, Comoviruses, Geminiviruses and Tobamoviruses. *Tobacco mosaic virus* (TMV) was the first virus engineered as a deconstructed vector system because of its high number of capsid protein (CP) copies (2130 copies) and the relatively simple organization of its genome. The TMV expression system is composed of two modules, one containing the replicase and the other containing the site for foreign gene integration. TMV has been used successfully for the production of several proteins of interest. The expression of vectors has been improved by genetic engineering with modifications of transcript splicing sites, codon usage patterns and insertion of introns into coding sequences [16]. Human papillomavirus (HPV) E7 protein has been generated in plants with TMV vectors [83]. Influenza M2e epitope has also been produced in plants thanks to TMV [54]. Others examples that illustrate the use of TMV with the production of the broadly neutralizing antibody against HIV-1 [84] or vaccines against cholera, influenza virus and plague have also been generated [55].

*Potato Virus X* (PVX), a flexuous, rod-shaped virus containing a plus-sense RNA molecule, has also been engineered extensively as an expression vector for biopharmaceutical production. The capsid protein gene and a triple gene block whose products are responsible for PVX movement are targeted to engineer deconstructed PVX vectors. PVX has been used to express full-length proteins, fusion proteins and epitopes for medical purposes [50, 85]. More recently, PVX nanoparticles have been demonstrated to block tumor progression in animal models [51, 86, 87].

The Comovirus *Cowpea mosaic virus* (CPMV) is an icosahedral virus of 30 nm in diameter. The genome of CPMV is bipartite, and its RNA-2 is the principal component used for expression vector development. CPMV has been utilized extensively in antigenic presentation and full-length protein expression as part of a fusion protein that can undergo proteolytic cleavage to release the therapeutic protein [88]. Medicago, Inc. (Durham, NC, USA) used the CPMV vector to generate VLPs carrying influenza virus HA antigens that protect against lethal viral challenge in animal models [89]. The CPMV deconstructed vector system pEAQ [90] allows the expression of foreign proteins without the need for viral replication. It thus enhances protein translation without viral cycle progression, which could have a negative impact on the stability of foreign gene integration. The resulting vector provides a substantial increase in foreign protein production [91, 92].

Besides RNA viruses that have been widely exploited as viral expression vector, there is a rise of single strand DNA (ssDNA) virus exploitation as viral vector for potential expression of a various single or even multiple proteins of interest in a wide range of plant families. The use of vacuum or other infiltration of whole plants by *Agrobacterium tumefaciens* suspensions has allowed the development of a set of expression vectors that rival the deconstructed RNA virus vectors in their yield and application, with some potential advantages over the latter that still need to be explored [58]. Recently, a novel ssDNA vector serie named pRIC has been developed based on the geminivirus *Bean yellow dwarf virus* (BeYDV) by the Rybicki group in South Africa. This ssDNA expression vector have been exploited to address African health concerns like HIV or HPV [59].

The technology of viral vectors based expression is more and more mastered; it allows nowadays the production of complex proteins. It has also opened up to new horizons such as the exploitation of viral vectors for the fight against cancers. Yet the developing African countries remains on the sidelines of these technologies, which would be undoubtedly a ready-made solution to the health problems that undermine this part of the world. In the past, vaccines produced through other expression systems have not had a positive impact in developing African countries. Those needing the most these product, populations of remote, impoverished regions, could not access vaccines and other biopharmaceutical proteins. This is mainly due to the high costs estimated for a successful immunization strategy and the absence of accessible infrastructure for vaccine production, distribution and delivery [17].

All these difficulties are reduced in the case of the expression of the proteins of interest in plants through viral expression vectors. Indeed, plant systems have the capacity to produce properly folded proteins at low cost, making them suitable expression systems for poor nations [93]. In addition, plants possess other advantages as expression systems; they do not harbor mammalian pathogens and, in certain instances, the expressed proteins can undergo similar posttranslational modifications to their mammalian counterparts. Pharmaceuticals derived from plants can easily be purified or, in specific cases, merely partially purified prior to oral administration. Plant systems provide an optimal delivery vehicle that prevents or delays the digestive hydrolysis of vaccine antigens. A protein drug delivery concept allowing the maintenance of folding, assembly and disulfide bonds and therapeutic efficacy for 2–3 years when stored at ambient temperature has been described [84, 94, 95].

The level of knowledge of the exploitation of plants as bioreactors and, more particularly, amplicon technology is such that it could be accessible for developing Africa. Several preliminary works based on plant and viral vector exploitation have already begun to target the diseases found in developing countries, such as NTDs, HBV (Hepatitis B virus), cancers, malaria and HIV.

### HIV

A safe and effective preventative vaccine is urgently needed to curb the HIV pandemic. Several strategies and candidates have been considered to achieve HIV-efficient vaccines [2, 96]. Paradigms have been shifting based on the results of previous clinical trials. First, the viral envelope was targeted as the best candidate to generate an immunogenic response, but this strategy has been abandoned because viral variability represents a barrier to obtaining a broad immune response [97, 98]. Attempts then turned to polyvalent vaccines targeting both the structural and regulatory proteins of HIV. A clinical evaluation of a multicomponent vaccine containing recombinant envelope surface glycoprotein (gp120) and a fusion protein between two regulatory viral proteins (Nef-Tat) was performed in uninfected human volunteers [99]. Moreover, the effects of a genetic vaccine combining both structural (Gag/, Env) and regulatory (Rev, Tat, Nef) viral proteins were evaluated in macaques developing Simian immunodeficiency virus (SIV) infection, an AIDS-like disease comparable to HIV infection in humans [100, 101]. These previous works lead to the conclusion that multiprotein vaccines induce robust vaccine-induced immunity that is able to abrogate viral replication. No multicomponent HIV candidate has yet been produced in plants, but several successes in HIV-derived protein expression have already been reported in plants [31, 102, 103]. Moreover, it has been demonstrated that the structural and regulatory proteins of HIV can be successfully expressed in plants by either stable transformation or transient expression systems [96, 104]. gp120 and gp41 from HIV envelope protein are HIV structural protein epitopes that target many approaches to produce HIV vaccines. Multiepitope chimeric proteins with sequences from gp120 (C4 and V3) and four variants of the epitope from gp41 have been produced in moss plants and elicit an immunogenic response when subcutaneously administered in mice [105]. The production of HIV regulatory proteins (Tat and Nef) has been reported in plant-based expression systems. The entire sequence of the HIV Tat protein was transiently expressed in *Nicotiana benthamiana* and spinach. The recombinant Tat produced in these plant systems allowed the systemic production of anti-HIV antibodies when it was used as a vaccine to immunize mice [106]. Nef protein exhibiting antigenic epitopes has been transiently expressed in the protoplasts of *Nicotiana tabacum* [107]. Finally, it has been shown that plant-derived vaccines are suitable for booster vaccines, particularly where the number of doses needed is important and when reinfection occurs in the population, as is the case for HIV in poor countries [2]. With the possibility of oral vaccination delivery using plant cells, this field opens up new possibilities for HIV vaccine production in plants.

### Malaria

RTS,S/AS01 (trade name Mosquirix), a recombinant protein-based malaria vaccine has been approved for use by European regulators in July 2015 [108, 109]. Several proteins from different stages of the malaria parasite life cycle have been studied as vaccine candidates. Plants and viral expression vectors have greatly contributed to the advanced search for an effective vaccine against malaria. Indeed, vaccines based upon key malaria immunogens have been generated in plants and have been demonstrated to be effective in inducing an immune response in mice. Plant codon optimization and the deconstructed TMV-based transient expression system allowed the expression of antigens against the asexual blood stage of plasmodium, such as *Plasmodium yoelii* merozoite surface protein 1 (PyMSP1), *P. yoelii* merozoite surface protein 4/5 (PyMSP4/5), and *P. falciparum* merozoite surface protein 1 (PfMSP1), at satisfactory yields [64, 110, 111]. Some of these antigens produced in plants are able to elicit an immune response by intraperitoneal or oral vaccination in a murine model [64, 110–113]. Plant-codon-optimized forms of the genes of interest combined with the TMV-based expression system have been shown to allow an increase of up to 100-fold compared to the transgenic *Nicotiana benthamiana* system [64, 110].

### NTDs: rabies

Because the production of pharmaceuticals against NTDs is not economically profitable for international pharmaceutical companies, plants represent an alternative choice. The World Health Organization (WHO), in an effort to reduce rabies burden, has chosen a plant-based expression system to procure and deliver safe and efficacious rabies vaccines in countries with a crucial need [114].

Rabies surface glycoprotein (G-protein) is the most important protein involved in viral pathogenesis. It has been recognized as the major protective antigen [115]. Several research groups have produced this glycoprotein in recombinant yeast or insect cells or in transgenic plants. The immunogenicity of the recombinant protein has been found to be variable in the diverse tested systems [116–120]. This may be due to the structural complexity of the rabies virus glycoprotein, which carries two N-linked oligosaccharide branches [121]. A plant-based expression system capable of this type of posttranslational modification is suitable for rabies G-protein production. As a pioneering plant-based work, tomato plants were engineered to express the G-protein from its nuclear genome. The protein was successfully immunoprecipitated and detected by Western blot from leaves and fruit [23]. Later, Yusibov and collaborators [23] reported the expression of an antigenic protein designated CPDrg24 (epitopes from rabies glycoprotein and nucleoprotein) fused with *Alfalfa mosaic virus* (AMV) capsid protein (CP). This chimeric protein has been successfully expressed in infected plants, yielding viral particles that were successfully purified from infected plant tissue and directly used to immunize mice. This work demonstrated that plant-produced rabies virus antigen was capable of inducing an immune response in mice in an adjuvant-free system [122].

### Emerging diseases

In addition to NTDs, there are new emerging diseases that also threaten poor nations. Ebola virus is a recently identified filovirus that causes hemorrhagic fever in both humans and nonhuman primates [4]. Ebola virus is also considered to be a potential biological threat in the hands of terrorists, as no effective vaccine exists yet [123]. Plants could be used for the rapid production of vaccines that might be necessary for protection against rapid Ebola outbreaks or bioterrorism. Investigations by means of plant biotechnology methods using tobacco have resulted in an experimental drug named ZMapp consisting of three monoclonal antibodies against the surface glycoprotein of Ebola virus responsible for binding and entry into host cells. ZMapp was used to treat two missionaries in Liberia, allowing them to recover from severe Ebola infection [124]. Another vaccine against Ebola virus disease, called Ebola Immunogenic Complex (EIC), was successfully transiently expressed in *Nicotiana benthamiana* [125]. This Ebola vaccine has also been generated in lettuce, a plant that does not contain the same levels of phenolic and toxic alkaloids as *Nicotiana* species and thus is more suitable for administration [126]. The use of lettuce is attractive because plant production can be readily upscaled, making it a feasible method for generating and stockpiling large amounts of inexpensive vaccine protein that could be worked up in the event of a terrorist attack.

Plants have also been useful in the production of an antibody against dengue fever virus, another filovirus causing tremendous and widespread damage in developing countries. A TMV-based transient expression system has been used to produce a dengue-virus-derived antigen in plant cells that exhibits appropriate antigenicity and immunogenicity [127].

### HPV and hepatitis

Plant-based expression systems and viral expression vectors represent a versatile technology allowing the expression of several proteins displaying vaccine properties against diverse diseases, such as viral diseases, that may eventually lead to cancer. Human papilloma viruses (HPVs) represent the main cause of cancer-related death for women in developing countries and the third leading cause of cancer-related death among women worldwide [128]. 120 HPV genotypes have been identified. Forty of them are able to infect the anogenital tract and are categorized into low and high risk HPV on the basis of their capacity to induce malignancy [129–132]. Thirteen HPV genotypes are categorized as high-risk (HR) because of their etiologic contribution to the development of cervical or other genital cancers [130, 133]. Currently available bivalent and quadrivalent vaccines target only HPV 16 and 18, leading causes of cervical cancer worldwide. The nonavalent vaccine target additional HR genotypes (HPV 16, 18, 31, 33, 45, 52, and 58) [134–136]. The genotypes targeted by these vaccines are not the most common circulating in some African regions. Some HR HPV genotypes not targeted by existing vaccines have been reported in African population [134]. HPV infection varies by region. Some studies have reported in Burkina Faso (Ouagadougou) other HR -HPV genotypes different from HPV 16 and HPV 18 (29.4% of HPV 35) [134, 137–140]. The situation seems to be the same in other countries of the subregion: Benin (Parakou) (38% of HPV 39) [141, 142]; Ivory Coast (Abidjan) (17.5% of HPV 35) [143]. HPV 16 is less frequent in Sub-Saharan Africa than in the rest of the world [144]. One of the main genotypes targeted by vaccines HPV 16 has been declared absent during a study in Benin [141] while the two genotypes HPV 16 and 18 were marginal during a study in Burkina [145]. Multiple infections of HPV-HR were also observed in 78.03% of infected women [145]. The three prophylactic vaccines listed above are VLP vaccine, based on the immunodominant L1 major capsid protein and have been shown to be effective in preventing cervical disease [135, 136]. However, these vaccines are expensive and type-specific reducing the protective effect for particularly low-resource countries. Hence, there is a need for next-generation HPV vaccines that broadly target oncogenic HPV types, at reduced cost particularly in developing countries suffering from cervical cancer [146]. The L2 protein is conserved across different strains of HPV. Anti-L2 antibodies can neutralize a broad range of mucosal and cutaneous HPVs [147, 148] suggesting that a L2 vaccine could address the type-restrictive efficacy of L1 vaccines. Next-generation vaccines using L2 peptides have been investigated to generate more cross-protective responses [149]. Several groups have reported the successful production of papillomavirus L1 capsid proteins in plants, both transgenic and transient expression of L1 has been done [38, 150–156]. Plant based expression of HPV vaccine combine to the new strategies focusing on HPV L2 peptide could be a potent way to achieve affordable HPV vaccine production for developing world.

Viral hepatitis is the most common causative agent of inflammation and damage of the liver. Hepatitis A, B or C viruses frequently cause viral hepatitis. While Hepatitis A Virus (HAV) causes the least amount of liver damage, Hepatitis B Virus (HBV) and Hepatitis C Virus (HCV) can lead to chronic liver disease and cancer. These viruses are found worldwide, but predominate in developing countries. An efficacious vaccine exists for Hepatitis A and B, but not for hepatitis C [157]. Vaccine target for HCV such as HCV surface protein and envelope protein-2 (E2) are highly variable, making the eradication of the virus difficult [158]. T Lymphocyte (CTL) response specific to HCV epitopes is the central immune mechanism characterizing the body’s defense against HCV disease [159–161]. Plant based pharmaceutical can play a key role to reduce HCV burden in developing world. In fact, VLPs derived from plant viruses can be used to express immunogenic epitopes of human viruses that stimulate strong T- and B-cell response. Papaya Mosaic Virus capsid protein carrying at the C-terminus Hepatitis C Virus E2 epitope induced humoral and cell-mediated immune responses [52].

Hepatitis B virus (HBV) is a worldwide disease that represents a significant burden on developing countries. Yeast-derived subunit vaccine was generated [24]. This vaccine is effective and contributes to reduce the HBV burden in the world. However, one could expect the establishment of cost effective vaccine against this disease through plant-based vaccines. Some success have been reported for adjuvant free oral transgenic plant-based HBV vaccines. Transgenic potato tubers expressing the surface antigen of HBV, HBsAg, were fed to individual volunteers. The vast majority of patients were able to produce a strong IgG (immunoglobulines G) titer in their blood serum [24]. This was the first proof-of-concept for the production of an immune response based upon the oral consumption of a vaccine antigen produced in a food crop plant. The same attempt has been made with lettuce, an attractive crop for producing an orally administered antigen [162]. The dominant HBV genotype in West Africa, genotype E, is not the same as those found elsewhere [163, 164]. As with HPV, it will be necessary to develop specific vaccines for West African HBV genotypes. Based on the previous works mentioned above, one could speculate that plant-virus-based biosystems would be particularly well adapted to respond to these specific African HBV genotypes.

### Obstacles and solutions to the effectiveness of viral expression vectors in Africa

Plant-based pharmaceutics and viral expression vectors represent a boon to resolve the health problems of the population of developing Africa. However, this technology is mainly applied by northern firms that are not economically interested in pharmaceutical production for diseases present in LDCs. In Africa, most countries that are involved in biotech activities are still at the level of tissue culture applications and they are generally limited to genetic engineering [165, 166]. Some production of therapeutics through genetic engineering has been reported in Tunisia [167, 168]. South Africa is the leader and has secured a seat for Africa in the world’s plant biotech field [169]. The Biopharming Research Unit of Edward Rybicki from the University of Cape Town has pioneered plant-made pharmaceutical production in Africa. The Rybicki’s group has developed its own proprietary virus-derived ssDNA vector for the expression of foreign proteins in plants [58, 59, 170]. This viral expression vector, based on BeYDV, was used to generate a candidate vaccine for human papillomavirus-16 (HPV-16) based on capsid protein L1, as well as a vaccine for the HIV-1 type C p24 antigen based on the Gag protein [58, 59]. Despite the efforts of this group, Africa accounts for less than 1% of the world Plant molecular farming centers [171].

The rest of Africa, especially developing Africa, is on the sidelines of this technological advance for a number of reasons. The expertise and the infrastructure necessary for the implementation of this technology are absent in these countries. In addition, the viral expression vectors that are key tools for the efficient production of therapeutics are patented, limiting their exploitation to large northern industrial firms. Other major obstacles to the implementation of plant bioreactors relate to biosafety concerns in these states. Moreover, the institutional frameworks for legislation on this type of product are poorly defined. The populations concerned must also be addressed, because they are generally resistant to biotech products using genetic engineering.

Plant viral vector technology could be a platform allowing developing nations to grow their own pharmaceutical industries. This effect could be long-lasting only if politicians adopt laws allowing LDCs to freely acquire and use virus constructs. This will necessarily go through the development of virus constructs based on African viral resources by African researchers. Excellent candidate viruses already exist, such as RNA viruses from the sobemovirus group, such as *Rice yellow mottle virus* (RYMV) and *Imperata yellow mottle virus* (IYMV), and DNA virus belonging to the group of begomoviruses, such as *Pepper yellow vein Mali virus* (PepYVMLV). Sobemoviruses present the advantage of having a small genome with just five open reading frames. Research groups from south Saharan Africa, especially those from Burkina Faso and their northern partners, already have a solid background in these three viruses [172–176]. Development of infectious clones working on *Nicotiana* species and natural virus host plants are ongoing (Lacombe, Tiendrébéogo, personal communication). These viral vectors could be used to treat human diseases such as malaria, HIV, Ebola, dengue, NTDs and animal diseases such as avian influenza (H5N1) but could also contribute to the production of antibodies for medical and research purposes.

Improvements in manufacturing infrastructure, financial support and the maturation of a regulatory framework surrounding plant-derived pharmaceuticals are all essential prerequisites for the implementation of this technology in the developing world. If these obstacles were overcome, many LDC health issues would find suitable solutions. Then, the plant-made pharmaceutical industry could become a reality for the developing world.

**Fig. 4:**
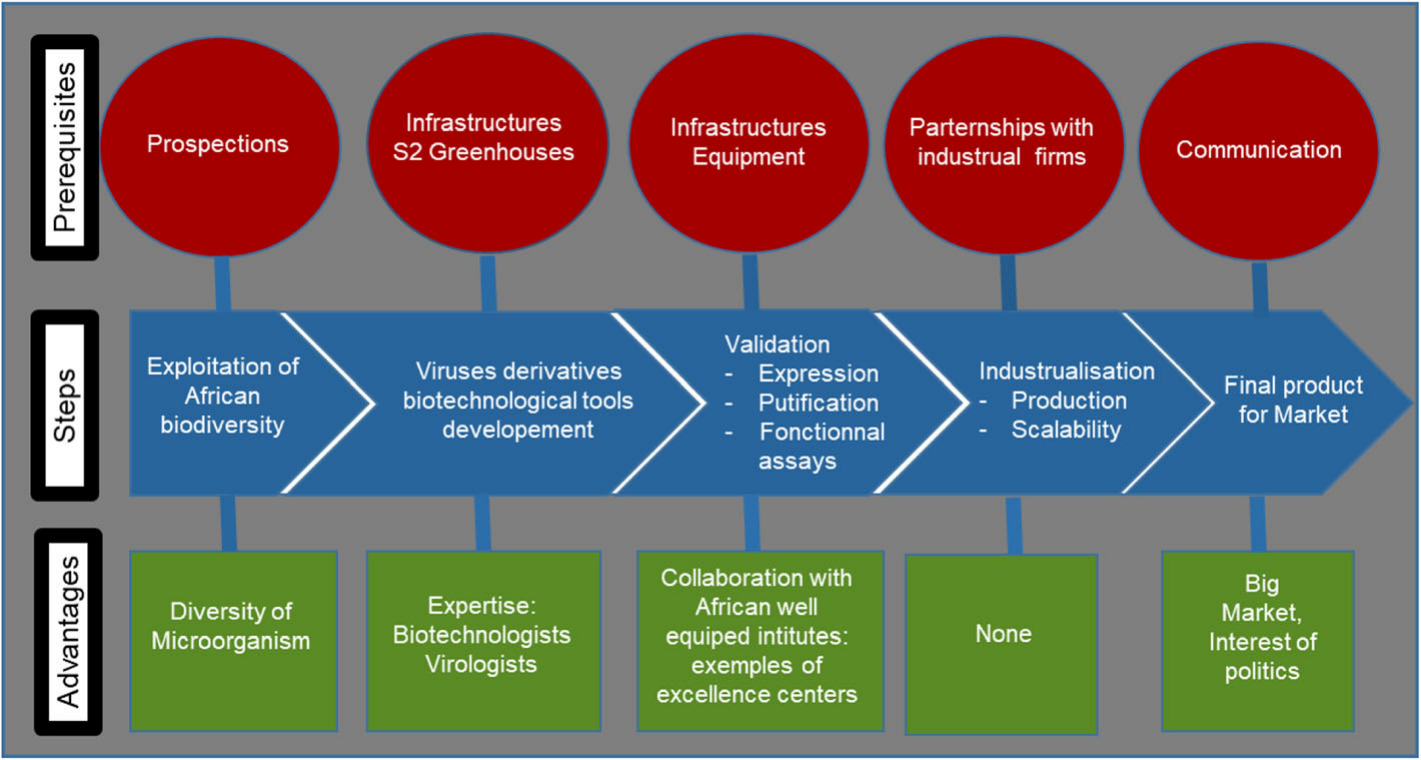
Schematic representation of the steps required for the development of plant-virus-based biotechnological tools in developing countries. For each step represented by the blue arrow, the prerequisites and benefits associated with the implementation in developing countries are shown in red and green, respectively

Such an implementation project requires solid infrastructure and expertise for both the medical and plant biological domains. This intersectional and multidisciplinary work implies different specialized institutions with deep knowledge of both plant science and medicine. Burkina Faso is one of the West African LDCs meeting these requirements. Indeed, Burkina Faso contains specialized structures for both aspects. The International Research Institute on Health Science (IRSS: Institut de Recherche en Science de la Santé) has strong expertise in parasites and pathogens such as malaria. LABIOGENE (Laboratoire de Biologie Moléculaire et de Génétique) is a molecular biology and genetic laboratory with crucial knowledge of genetic engineering for human health. On the plant side, the National Institute of Environment and Agronomy Research (INERA: Institut de l’Environnement et de Recherches Agricoles) displays a deep expertise on cultivated plants and their pathogens. In accordance with the “one health concept”, these three institutes would provide a complementary context to discover and develop plant viral expression vectors. IRSS and LABIOGENE are institutes with subregional missions recognized as centers of excellence of by the UEMOA (Union Economique et Monétaire Ouest Africaine). Experts on the plant side can benefit from the technical platforms of these two institutes to strengthen their activities in the “one health concept”, as the techniques used for human and animal diagnostics are similar to those used for experiments based on plants. Such collaborations would help reduce the costs of equipment and infrastructure. Furthermore, Africa has diverse biological resources that are underexploited. Indeed, some plants and pathogens endemic to Africa may be excellent candidates for viral amplicon/bioreactor technologies. African resources could then be exploited by and for Africans, as the Nagoya protocol advises [177]. Figure 4 is a schematic representation of the steps toward African plant-made medicines.

## Conclusion

Plant made therapeutics are safe with a reduced cost and thanks to viral vector they can be obtain in days rather months as at their beginning. By the past, poor resource communities could not afford medicines because of the production cost and logistic cost involved. Plant made therapeutics have been produced for major disease that prevail in these countries through either time-consuming transgenic approach or quick transient expression system using viral vectors. Most of these viral vectors that are patented can be used through win-win collaborations with research laboratories or private companies. To support plant-made therapeutics as a strategic means to overcome poor countries’ diseases, these countries should be equipped with their own viral constructs to provide them with the freedom to operate. Some plant viruses are present in Africa and have already been recognized as excellent candidates for viral amplicons made in and for Africa. This will be possible with the support of resource-poor countries’ governments through infrastructure, financial funding and the establishment of an efficient regulatory framework. Regarding the context, the implementation of plant-made biopharmaceuticals in the developing world is an ineluctable event. This would require new biotechnological tools for protein expression, such as new strong promoters, silencing suppressors or efficient viral expression vectors. Industrial firms should not avoid this field, as they have the opportunity to benefit from the advent of African viral-based biotechnological tool development.

## Data Availability

Not applicable.

## Abbreviations

2G12: HIV monoclonal antibody
Ag: Antigen
AIDS: Acquired immune deficiency syndrome
AMCV: *Artichoke mottle crinckle virus*
AMV: *Alfalfa mosaic virus*
BCTV: *Beet curly top virus*
BeYDV: *Bean Yellow drawf virus*
CMV: *Cucumber mosaic virus*
CP: Coat protein
CPDrg24: Epitopes from rabies glycoprotein and nucleoprotein
CPMV: *Cowpea mosaic virus*
DOX: Doxorubicin
E2: Envelope 2 protein
EIC: Ebola Immunogenic Complex
EVD: Ebola Virus Diseases
G protein: Surface glycoprotein
GAD65: Human glutamic acid decarboxylase
Gag: Group Antigens
GFP: Green fluorescent protein
GOI: Gene of interest
Gp120: Glycoprotein 120
HAV: Hepatitis A virus
HBsAg: Surface antigen of hepatitis B virus
HBV: Hepatitis B virus
HCRSV: *Hibiscus chlorotic ringspot virus*
HCV: Hepatitis C virus
HIV: Human immunodeficiency virus
HPV: Human papillomavirus
HPV16 L2: Human papillomavirus serotype 16 gene L2
HR: High risk
IgG: Immunoglobulines G
INERA: Institut de l’Environnement et Recherches Agricoles
IRSS: Institut de Recherche en Sciences de la Santé
IYMV: *Imperata yellow mottle virus*
LABIOGENE: Laboratoire de Biologie moléculaire et de Génétique
LDCs: Least developing countries
LFW: Leaf fresh weight
Nef: Negative Regulatory Factor
NTDs: Neglected tropical diseases
NVCP: Norwalk virus (NV) capsid protein
ORF: Open reading frame
PAA: Polyacrylic acid
PapMV: *Papaya mosaic potexvirus*
PepYVMLV: *Pepper yellow vein Mali virus*
PfMSP1: *Plasmodium falciparum* merozoite surface protein 1
PLD: PEGylated liposomal doxorubicin
PSA: Polystyrene sulfonic acid
PVX: *Potato Virus X*
PyMSP1: *Plasmodium yoelii* merozoite surface protein 1
PyMSP4/5: *Plasmodium yoelii* merozoite surface protein 4/5
RCNMV: *Red clover necrotic mosaic virus*
Rev.: regulator of expression of virion proteins
RYMV: *Rice yellow mottle virus*
SHMV: *Sun hemp mosaic virus*
SIV: *Simian immunodeficiency virus*
ssDNA: Single strand DNA
ssRNA: Single strand RNA
Tat: Transactivator of transcription
TBSV: *Tomato bushy stunt virus*
TMV: *Tobacco mosaic virus*
TSP: Total soluble proteins
TYDV: *Tobacco yellow dwarf virus*
UEMOA: Union Economique et Monétaire Ouest Africaine
VLPs: Virus Like Particles
WHO: World Health Organization
ZMapp: Ebola experimental drug name this way by Mapp biopharmaceutical Inc

## References

[1] UNAIDS DATA 2017. http://www.unaids.org/en/resources/documents/2017/2017_data_book. .

[2] Habibi P, Daniell H, Soccol CR, Grossi-de-Sa MF (2019). The potential of plant systems to break the HIV-TB link. Plant Biotechnol J.

[3] Updated_NTD_report2011_CS3_ok.indd. 2011;:25.

[4] Feldmann H, Jones S, Klenk H-D, Schnittler H-J (2003). Timeline: Ebola virus: from discovery to vaccine. Nat Rev Immunol.

[5] Ebola virus disease. World Health Organization. http://www.who.int/news-room/fact-sheets/detail/ebola-virus-disease. Accessed 10 Jul 2019.

[6] WHO | Malaria in pregnant women. WHO. http://www.who.int/malaria/areas/high_risk_groups/pregnancy/en/. Accessed 18 Jul 2019.

[7] Hefferon Kathleen (2017). Plant Virus Expression Vectors: A Powerhouse for Global Health. Biomedicines.

[8] Rybicki EP (2014). Plant-based vaccines against viruses. Virol J.

[9] Daniell Henry, Kulis Michael, Herzog Roland W. (2019). Plant cell-made protein antigens for induction of Oral tolerance. Biotechnology Advances.

[10] Ibrahim A, Odon V, Kormelink R (2019). Plant viruses in plant molecular pharming: toward the use of enveloped viruses. Front Plant Sci.

[11] Giddings G, Allison G, Brooks D, Carter A (2000). Transgenic plants as factories for biopharmaceuticals. Nat Biotechnol.

[12] MDG Report 2010 En 20100604 r14 Final.indd. Millenn Dev GOALS Rep. 2010:80.

[13] Daniell H, Streatfield SJ, Rybicki EP (2015). Advances in molecular farming: key technologies, scaled up production and lead targets. Plant Biotechnol J.

[14] Daniell H, Rai V, Xiao Y (2019). Cold chain and virus-free oral polio booster vaccine made in lettuce chloroplasts confers protection against all three poliovirus serotypes. Plant Biotechnol J.

[15] Lindbo JA (2007). TRBO: a high-efficiency tobacco mosaic virus RNA-based overexpression vector. Plant Physiol.

[16] Hefferon Kathleen (2014). Plant Virus Expression Vector Development: New Perspectives. BioMed Research International.

[17] Daniell H, Singh ND, Mason H, Streatfield SJ (2009). Plant-made vaccine antigens and biopharmaceuticals. Trends Plant Sci.

[18] Lee JH, Ko K (2017). Production of recombinant anti-Cancer vaccines in plants. Biomol Ther.

[19] Raskin I, Ribnicky DM, Komarnytsky S, Ilic N, Poulev A, Borisjuk N (2002). Plants and human health in the twenty-first century. Trends Biotechnol.

[20] Morens DM, Folkers GK, Fauci AS. The challenge of emerging and re-emerging infectious diseases 2004;430:8. 10.1038/nature02759 PMC7094993 15241422

[21] Xu K, Evans DB, Carrin G, Aguilar-Rivera AM, Musgrove P, Evans T (2007). Protecting Households From Catastrophic Health Spending. Health Aff (Millwood).

[22] Mason HS, Lam DM, Arntzen CJ (1992). Expression of hepatitis B surface antigen in transgenic plants. Proc Natl Acad Sci.

[23] McGarvey PB, Hammond J, Dienelt MM, Hooper DC, Fu ZF, Dietzschold B (1995). Expression of the rabies virus glycoprotein in transgenic tomatoes. Nat Biotechnol.

[24] Thanavala Y, Yang YF, Lyons P, Mason HS, Arntzen C (1995). Immunogenicity of transgenic plant-derived hepatitis B surface antigen. Proc Natl Acad Sci.

[25] Richter LJ, Thanavala Y, Arntzen CJ, Mason HS (2000). Production of hepatitis B surface antigen in transgenic plants for oral immunization. Nat Biotechnol.

[26] Wigdorovitz A, Carrillo C, Dus Santos MJ, Trono K, Peralta A, Gómez MC (1999). Induction of a protective antibody response to foot and mouth disease virus in mice following Oral or parenteral immunization with alfalfa transgenic plants expressing the viral structural protein VP1. Virology.

[27] Merlin M, Gecchele E, Capaldi S, Pezzotti M, Avesani L (2014). Comparative evaluation of recombinant protein production in different biofactories: the green perspective. Biomed Res Int.

[28] Sparrow P, Broer I, Hood E, Eversole K, Hartung F, Schiemann J (2013). Risk assessment and regulation of molecular farming - a comparison between Europe and US. Curr Pharm Des.

[29] Yao J, Weng Y, Dickey A, Wang K (2015). Plants as factories for human pharmaceuticals: applications and challenges. Int J Mol Sci.

[30] Breyer D, Goossens M, Herman P, Sneyers M (2009). Biosafety considerations associated with molecular farming in genetically modified plants. J Med Plants Res.

[31] Ahmad K (2014). Molecular farming: strategies, expression systems and bio-safety considerations. Czech J Genet Plant Breed.

[32] Kapila J, De Rycke R, Van Montagu M, Angenon G (1997). An agrobacterium-mediated transient gene expression system for intact leaves. Plant Sci.

[33] Porceddu A, Falorni A, Ferradini N, Cosentino A, Calcinaro F, Faleri C (1999). Transgenic plants expressing human glutamic acid decarboxylase (GAD65), a major autoantigen in insulin-dependent diabetes mellitus. Mol Breed.

[34] Zhang X, Buehner NA, Hutson AM, Estes MK, Mason HS (2006). Tomato is a highly effective vehicle for expression and oral immunization with Norwalk virus capsid protein. Plant Biotechnol J.

[35] Rosenberg Y, Sack M, Montefiori D, Forthal D, Mao L, Abanto SH (2013). Rapid High-Level Production of Functional HIV Broadly Neutralizing Monoclonal Antibodies in Transient Plant Expression Systems. PLoS One.

[36] Souza AC, Vasques RM, Inoue-Nagata AK, Lacorte C, Maldaner FR, Noronha EF (2013). Expression and assembly of Norwalk virus-like particles in plants using a viral RNA silencing suppressor gene. Appl Microbiol Biotechnol.

[37] Jones RM, Chichester JA, Manceva S, Gibbs SK, Musiychuk K, Shamloul M (2015). A novel plant-produced Pfs25 fusion subunit vaccine induces long-lasting transmission blocking antibody responses. Hum Vaccines Immunother.

[38] Zahin Maryam, Joh Joongho, Khanal Sujita, Husk Adam, Mason Hugh, Warzecha Heribert, Ghim Shin-je, Miller Donald M., Matoba Nobuyuki, Jenson Alfred Bennett (2016). Scalable Production of HPV16 L1 Protein and VLPs from Tobacco Leaves. PLOS ONE.

[39] Mardanova ES, Blokhina EA, Tsybalova LM, Peyret H, Lomonossoff GP, Ravin NV (2017). Efficient transient expression of recombinant proteins in plants by the novel pEff vector based on the genome of potato virus X. Front Plant Sci.

[40] Lacombe S, Bangratz M, Brizard J-P, Petitdidier E, Pagniez J, Sérémé D (2018). Optimized transitory ectopic expression of promastigote surface antigen protein in Nicotiana benthamiana , a potential anti-leishmaniasis vaccine candidate. J Biosci Bioeng.

[41] Chabeda A, van Zyl AR, Rybicki EP, Hitzeroth II. Substitution of human papillomavirus type 16 L2 neutralizing epitopes into L1 surface loops: the effect on virus-like particle assembly and immunogenicity. Front Plant Sci. 2019;10. 10.3389/fpls.2019.00779. 10.3389/fpls.2019.00779 PMC6597877 31281327

[42] Johansen LK (2001). Silencing on the spot. Induction and suppression of rna silencing in the agrobacterium-mediated transient expression system. PLANT Physiol.

[43] Brodersen P, Voinnet O (2006). The diversity of RNA silencing pathways in plants. Trends Genet.

[44] Incarbone M, Dunoyer P (2013). RNA silencing and its suppression: novel insights from in planta analyses. Trends Plant Sci.

[45] Lombardi R, Circelli P, Villani M, Buriani G, Nardi L, Coppola V (2009). High-level HIV-1 Nef transient expression in Nicotiana benthamiana using the P19 gene silencing suppressor protein of artichoke mottled Crinckle virus. BMC Biotechnol.

[46] Garabagi F, Gilbert E, Loos A, McLean MD, Hall JC (2012). Utility of the P19 suppressor of gene-silencing protein for production of therapeutic antibodies in *Nicotiana* expression hosts. Plant Biotechnol J.

[47] Gleba Y, Klimyuk V, Marillonnet S (2007). Viral vectors for the expression of proteins in plants. Curr Opin Biotechnol.

[48] Yusibov V, Shivprasad S, Turpen TH, Dawson W, Koprowski H. Plant Viral Vectors Based on Tobamoviruses. In: Hammond J, McGarvey P, Yusibov V, editors. Plant Biotechnology. Berlin, Heidelberg: Springer Berlin Heidelberg; 2000. p. 81–94. doi:10.1007/978-3-642-60234.

[49] Turpen TH (1999). Tobacco mosaic virus and the virescence of biotechnology. Philos Trans R Soc B Biol Sci.

[50] Uhde-Holzem K, Schlösser V, Viazov S, Fischer R, Commandeur U (2010). Immunogenic properties of chimeric potato virus X particles displaying the hepatitis C virus hypervariable region I peptide R9. J Virol Methods.

[51] Mardanova ES, Kotlyarov RY, Kuprianov VV, Stepanova LA, Tsybalova LM, Lomonosoff GP, et al. Rapid high-yield expression of a candidate influenza vaccine based on the ectodomain of M2 protein linked to flagellin in plants using viral vectors. BMC Biotechnol. 2015;15. 10.1186/s12896-015-0164-6. 10.1186/s12896-015-0164-6 PMC4446962 26022390

[52] Denis J, Majeau N, Acosta-Ramirez E, Savard C, Bedard M-C, Simard S (2007). Immunogenicity of papaya mosaic virus-like particles fused to a hepatitis C virus epitope: evidence for the critical function of multimerization. Virology.

[53] Denis J, Acosta-Ramirez E, Zhao Y, Hamelin M-E, Koukavica I, Baz M (2008). Development of a universal influenza a vaccine based on the M2e peptide fused to the papaya mosaic virus (PapMV) vaccine platform. Vaccine.

[54] Noris E, Poli A, Cojoca R, Rittà M, Cavallo F, Vaglio S (2011). A human papillomavirus 8 E7 protein produced in plants is able to trigger the mouse immune system and delay the development of skin lesions. Arch Virol.

[55] Musiychuk K, Stephenson N, Bi H, Farrance CE, Orozovic G, Brodelius M (2007). A launch vector for the production of vaccine antigens in plants: plant-produced vaccines. Influenza Other Respir Viruses.

[56] Liu Z, Kearney CM (2010). A tobamovirus expression vector for agroinfection of legumes and Nicotiana. J Biotechnol.

[57] Steinmetz NF, Cho C-F, Ablack A, Lewis JD, Manchester M (2011). Cowpea mosaic virus nanoparticles target surface vimentin on cancer cells. Nanomed.

[58] Rybicki EP, Martin DP (2014). Virus-derived ssDNA vectors for the expression of foreign proteins in plants. Curr Top Microbiol Immunol.

[59] Regnard GL, Halley-Stott RP, Tanzer FL, Hitzeroth II, Rybicki EP (2010). High level protein expression in plants through the use of a novel autonomously replicating geminivirus shuttle vector. Plant Biotechnol J.

[60] Chung HY, Lee HH, Kim KI, Chung HY, Hwang-Bo J, Park JH (2011). Expression of a recombinant chimeric protein of hepatitis a virus VP1-fc using a replicating vector based on beet curly top virus in tobacco leaves and its immunogenicity in mice. Plant Cell Rep.

[61] Dugdale B, Mortimer CL, Kato M, James TA, Harding RM, Dale JL (2013). In plant activation: an inducible, Hyperexpression Platform for Recombinant Protein Production in Plants. Plant Cell.

[62] Kagale S, Uzuhashi S, Wigness M, Bender T, Yang W, Borhan MH, et al. TMV-gate vectors: gateway compatible tobacco mosaic virus based expression vectors for functional analysis of proteins. Sci Rep. 2012;2. 10.1038/srep00874. 10.1038/srep00874 PMC3500846 23166857

[63] Turpen T, Reinl S, Charoenvit Y, Hoffman S, Fallarme V, Grill L (1995). Malarial epitopes expressed on the surface of recombinant tobacco mosaic-virus. Bio-Technol.

[64] Webster DE, Wang L, Mulcair M, Ma C, Santi L, Mason HS (2009). Production and characterization of an orally immunogenic *Plasmodium* antigen in plants using a virus-based expression system. Plant Biotechnol J.

[65] Gleba Y, Marillonnet S, Klimyuk V (2004). Engineering viral expression vectors for plants: the ‘full virus’ and the ‘deconstructed virus’ strategies. Curr Opin Plant Biol.

[66] Avesani L, Marconi G, Morandini F, Albertini E, Bruschetta M, Bortesi L (2007). Stability of potato virus X expression vectors is related to insert size: implications for replication models and risk assessment. Transgenic Res.

[67] Gleba Y, Klimyuk V, Marillonnet S (2005). Magnifection?A new platform for expressing recombinant vaccines in plants. Vaccine.

[68] Liu L, Lomonossoff GP (2002). Agroinfection as a rapid method for propagating cowpea mosaic virus-based constructs. J Virol Methods.

[69] J√^o^ttner G, Baulcombe DC, Fedorkin ON, Schiemann J, Atabekov JG, Morozov SYu. Complementation of a potato virus X mutant mediated by bombardment of plant tissues with cloned viral movement protein genes. J Gen Virol. 1997;78:2077. 10.1099/0022-1317-78-8-2077 9267010

[70] Ziegler-Graff V, Guilford PJ, Baulcombe DC (1991). Tobacco rattle virus RNA-1 29K gene product potentiates viral movement and also affects symptom induction in tobacco. Virology.

[71] Deom CM, Oliver MJ, Beachy RN (1987). The 30-kilodalton gene product of tobacco mosaic virus potentiates virus movement. Science.

[72] Kaplan IB, Shintaku MH, Li Q, Zhang L, Marsh LE, Palukaitis P (1995). Complementation of virus movement in transgenic tobacco expressing the cucumber mosaic virus 3a gene. Virology.

[73] van der Kuyl AC, Neeleman L, Bol JF (1991). Complementation and recombination between alfalfa mosaic virus RNA3 mutants in tobacco plants. Virology.

[74] Le DHT, Hu H, Commandeur U, Steinmetz NF (2017). Chemical addressability of potato virus X for its applications in bio/nanotechnology. J Struct Biol.

[75] Saunders K, Sainsbury F, Lomonossoff GP (2009). Efficient generation of cowpea mosaicvirus empty virus-like particles by the proteolytic processing of precursors in insect cells and plants. Virology.

[76] Gonzalez MJ, Plummer EM, Rae CS, Manchester M (2009). Interaction of cowpea mosaic virus (CPMV) nanoparticles with antigen presenting cells in vitro and in vivo. PLoS One.

[77] Ren Y, Wong SM, Lim L-Y (2007). Folic acid-conjugated protein cages of a plant virus: a novel delivery platform for doxorubicin. Bioconjug Chem.

[78] Franke CE, Czapar AE, Patel RB, Steinmetz NF (2018). Tobacco mosaic virus-delivered Cisplatin restores efficacy in platinum-resistant ovarian Cancer cells. Mol Pharm.

[79] Babin C, Majeau N, Leclerc D (2013). Engineering of papaya mosaic virus (PapMV) nanoparticles with a CTL epitope derived from influenza NP. J Nanobiotechnology.

[80] Lebel M-È, Chartrand K, Tarrab E, Savard P, Leclerc D, Lamarre A (2016). Potentiating Cancer immunotherapy using papaya mosaic virus-derived nanoparticles. Nano Lett.

[81] Madden AJ, Oberhardt B, Lockney D, Santos C, Vennam P, Arney D (2017). Pharmacokinetics and efficacy of doxorubicin-loaded plant virus nanoparticles in preclinical models of cancer. Nanomed.

[82] Narayanan KB, Han SS (2017). Icosahedral plant viral nanoparticles - bioinspired synthesis of nanomaterials/nanostructures. Adv Colloid Interf Sci.

[83] Massa S, Franconi R, Brandi R, Muller A, Mett V, Yusibov V (2007). Anti-cancer activity of plant-produced HPV16 E7 vaccine. Vaccine.

[84] Hamorsky KT, Grooms-Williams TW, Husk AS, Bennett LJ, Palmer KE, Matoba N (2013). Efficient single tobamoviral vector-based bioproduction of broadly neutralizing anti-HIV-1 monoclonal antibody VRC01 in Nicotiana benthamiana plants and utility of VRC01 in combination microbicides. Antimicrob Agents Chemother.

[85] Mohammadzadeh S, Roohvand F, Memarnejadian A, Jafari A, Ajdary S, Salmanian A-H (2016). Co-expression of hepatitis C virus polytope–HBsAg and p19-silencing suppressor protein in tobacco leaves. Pharm Biol.

[86] Shukla S, Ablack AL, Wen AM, Lee KL, Lewis JD, Steinmetz NF (2013). Increased tumor homing and tissue penetration of the filamentous plant viral nanoparticle *Potato virus X*. Mol Pharm.

[87] Demurtas OC, Massa S, Illiano E, De Martinis D, Chan PKS, Di Bonito P, et al. Antigen production in plant to tackle infectious diseases flare up: the case of SARS. Front Plant Sci. 2016;7. 10.3389/fpls.2016.00054. 10.3389/fpls.2016.00054 PMC4742786 26904039

[88] Sainsbury F, Lavoie P-O, D’Aoust M-A, Vézina L-P, Lomonossoff GP (2007). Expression of multiple proteins using full-length and deleted versions of cowpea mosaic virus RNA-2. Plant Biotechnol J.

[89] Mardanova ES, Kotlyarov RY, Kuprianov VV, Stepanova LA, Tsybalova LM, Lomonossoff GP (2016). High immunogenicity of plant-produced candidate influenza vaccine based on the M2e peptide fused to flagellin. Bioengineered..

[90] Sainsbury F, Thuenemann EC, Lomonossoff GP (2009). pEAQ: versatile expression vectors for easy and quick transient expression of heterologous proteins in plants. Plant Biotechnol J.

[91] Montague NP, Thuenemann EC, Saxena P, Saunders K, Lenzi P, Lomonossoff GP (2011). Recent advances of cowpea mosaic virus-based particle technology. Hum Vaccin.

[92] Meshcheriakova YA, Saxena P, Lomonossoff GP (2014). Fine-tuning levels of heterologous gene expression in plants by orthogonal variation of the untranslated regions of a nonreplicating transient expression system. Plant Biotechnol J.

[93] Wang L, Kedzierski L, Wesselingh SL, Coppel RL (2003). Oral immunization with a recombinant malaria protein induces conformational antibodies and protects mice against lethal malaria. Infect Immun.

[94] Herzog RW, Nichols TC, Su J, Zhang B, Sherman A, Merricks EP (2017). Oral tolerance induction in hemophilia B dogs fed with Transplastomic lettuce. Mol Ther.

[95] Su J, Zhu L, Sherman A, Wang X, Lin S, Kamesh A (2015). Low cost industrial production of coagulation factor IX bioencapsulated in lettuce cells for oral tolerance induction in hemophilia B. Biomaterials.

[96] Rosales-Mendoza S, Rubio-Infante N, Govea-Alonso DO, Moreno-Fierros L (2012). Current status and perspectives of plant-based candidate vaccines against the human immunodeficiency virus (HIV). Plant Cell Rep.

[97] Walker BD, Burton DR (2008). Toward an AIDS vaccine. Science.

[98] Fauci AS, Johnston MI, Dieffenbach CW, Burton DR, Hammer SM, Hoxie JA (2008). HIV vaccine research: the way forward. Science.

[99] Goepfert PA, Tomaras GD, Horton H, Montefiori D, Ferrari G, Deers M (2007). Durable HIV-1 antibody and T-cell responses elicited by an adjuvanted multi-protein recombinant vaccine in uninfected human volunteers. Vaccine.

[100] Voss G, Manson K, Montefiori D, Watkins DI, Heeney J, Wyand M (2003). Prevention of disease induced by a partially heterologous AIDS virus in rhesus monkeys by using an Adjuvanted multicomponent protein vaccine. J Virol.

[101] Maggiorella MT, Sernicola L, Crostarosa F, Belli R, Pavone-Cossut MR, Macchia I (2007). Multiprotein genetic vaccine in the SIV-Macaca animal model: a promising approach to generate sterilizing immunity to HIV infection. J Med Primatol.

[102] Habibi P, Soccol CR, O’Keefe BR, Krumpe LRH, Wilson J, de Macedo LLP (2018). Gene-silencing suppressors for high-level production of the HIV-1 entry inhibitor griffithsin in Nicotiana benthamiana. Process Biochem.

[103] Ataie Kachoie E, Behjatnia SAA, Kharazmi S (2018). Expression of human immunodeficiency virus type 1 (HIV-1) coat protein genes in plants using cotton leaf curl Multan betasatellite-based vector. PLoS One.

[104] Marusic C, Vitale A, Pedrazzini E, Donini M, Frigerio L, Bock R (2009). Plant-based strategies aimed at expressing HIV antigens and neutralizing antibodies at high levels. Nef as a case study Transgenic Res.

[105] Orellana-Escobedo L, Rosales-Mendoza S, Romero-Maldonado A, Parsons J, Decker EL, Monreal-Escalante E (2015). An Env-derived multi-epitope HIV chimeric protein produced in the moss Physcomitrella patens is immunogenic in mice. Plant Cell Rep.

[106] Karasev AV, Foulke S, Wellens C, Rich A, Shon KJ, Zwierzynski I (2005). Plant based HIV-1 vaccine candidate: tat protein produced in spinach. Vaccine.

[107] Barbante A, Irons S, Hawes C, Frigerio L, Vitale A, Pedrazzini E (2008). Anchorage to the cytosolic face of the endoplasmic reticulum membrane: a new strategy to stabilize a cytosolic recombinant antigen in plants. Plant Biotechnol J.

[108] Gosling Roly, von Seidlein Lorenz (2016). The Future of the RTS,S/AS01 Malaria Vaccine: An Alternative Development Plan. PLOS Medicine.

[109] Dobaño C, Ubillos I, Jairoce C, Gyan B, Vidal M, Jiménez A (2019). RTS,S/AS01E immunization increases antibody responses to vaccine-unrelated *Plasmodium falciparum* antigens associated with protection against clinical malaria in African children: a case-control study. BMC Med.

[110] Wang L, Webster DE, Campbell AE, Dry IB, Wesselingh SL, Coppel RL (2008). Immunogenicity of Plasmodium yoelii merozoite surface protein 4/5 produced in transgenic plants. Int J Parasitol.

[111] Ma C, Wang L, Webster DE, Campbell AE, Coppel RL (2012). Production, characterisation and immunogenicity of a plant-made Plasmodium antigen—the 19 kDa C-terminal fragment of Plasmodium yoelii merozoite surface protein 1. Appl Microbiol Biotechnol.

[112] Davoodi-Semiromi A, Schreiber M, Nalapalli S, Verma D, Singh ND, Banks RK (2010). Chloroplast-derived vaccine antigens confer dual immunity against cholera and malaria by oral or injectable delivery. Plant Biotechnol J.

[113] Lee C, Kim H-H, Mi Choi K, Won Chung K, Choi Y, Jang M (2011). Murine immune responses to a Plasmodium vivax-derived chimeric recombinant protein expressed in Brassica napus. Malar J.

[114] WHO | Deworming for Health and Development: Report of the third global meeting of the partners for parasite control. WHO. http://www.who.int/schistosomiasis/resources/WHO_CDS_CPE_PVC_2005.14/en/. Accessed 18 Sep 2019.

[115] Cox JH, Dietzschold B, Schneider LG. Rabies virus glycoprotein. II. Biological and serological characterization. Infect Immun. 1977;16:754. 10.1128/iai.16.3.754-759.1977 PMC421026 408269

[116] Batista FRX, Moraes ÂM, Büntemeyer H, Noll T (2009). Influence of culture conditions on recombinant Drosophila melanogaster S2 cells producing rabies virus glycoprotein cultivated in serum-free medium. Biologicals.

[117] Ben Azoun S, Belhaj AE, Göngrich R, Gasser B, Kallel H (2016). Molecular optimization of rabies virus glycoprotein expression in *Pichia pastoris*. Microb Biotechnol.

[118] Ramya R, Mohana Subramanian B, Sivakumar V, Senthilkumar RL, Sambasiva Rao KRS, Srinivasan VA (2011). Expression and Solubilization of insect cell-based rabies virus glycoprotein and assessment of its immunogenicity and protective efficacy in mice. Clin Vaccine Immunol.

[119] Yadav AS, Gahlot K, Gahlot GC, Asraf M, Yadav ML (2015). Microsatellite DNA typing for assessment of genetic variability in Marwari breed of Indian goat. Vet World.

[120] Tiwari S, Mishra DK, Roy S, Singh A, Singh PK, Tuli R (2009). High level expression of a functionally active cholera toxin B: rabies glycoprotein fusion protein in tobacco seeds. Plant Cell Rep.

[121] Shakin-Eshleman SH, Remaley AT, Eshleman JR, Wunner WH, Spitalnik SL (1992). N-linked glycosylation of rabies virus glycoprotein. Individual sequons differ in their glycosylation efficiencies and influence on cell surface expression. J Biol Chem.

[122] Yusibov V, Modelska A, Steplewski K, Agadjanyan M, Weiner D, Hooper DC (1997). Antigens produced in plants by infection with chimeric plant viruses immunize against rabies virus and HIV-1. Proc Natl Acad Sci.

[123] Strauss S (2014). Ebola research fueled by bioterrorism threat. Can Med Assoc J.

[124] Arntzen C (2015). Plant-made pharmaceuticals: from ‘edible vaccines’ to Ebola therapeutics. Plant Biotechnol J.

[125] Phoolcharoen W, Bhoo SH, Lai H, Ma J, Arntzen CJ, Chen Q (2011). Expression of an immunogenic Ebola immune complex in Nicotiana benthamiana: Ebola immune complex expression. Plant Biotechnol J.

[126] Lai H, He J, Engle M, Diamond MS, Chen Q (2012). Robust production of virus-like particles and monoclonal antibodies with geminiviral replicon vectors in lettuce: vaccine and antibody expression in lettuce. Plant Biotechnol J.

[127] Saejung W, Fujiyama K, Takasaki T, Ito M, Hori K, Malasit P (2007). Production of dengue 2 envelope domain III in plant using TMV-based vector system. Vaccine.

[128] Parkin DM, Pisani P, Ferlay J (1999). Estimates of the worldwide incidence of 25 major cancers in 1990. Int J Cancer.

[129] Yanofsky VR, Patel RV, Goldenberg G (2012). Genital warts: a comprehensive review. J Clin Aesthetic Dermatol.

[130] Steben M, Duarte-Franco E (2007). Human papillomavirus infection: epidemiology and pathophysiology. Gynecol Oncol.

[131] Gross G, Pfister H (2004). Role of human papillomavirus in penile cancer, penile intraepithelial squamous cell neoplasias and in genital warts. Med Microbiol Immunol (Berl).

[132] Thomas R, Steben M, Greenwald Z, Stutz M, Rodier C, DeAngelis F (2017). Recurrence of human papillomavirus external genital wart infection among high-risk adults in Montréal. Canada Sex Transm Dis.

[133] Walboomers JM, Jacobs MV, Manos MM, Bosch FX, Kummer JA, Shah KV (1999). Human papillomavirus is a necessary cause of invasive cervical cancer worldwide. J Pathol.

[134] Ouedraogo RA, Zohoncon TM, Guigma SP, Angèle Traore IM, Ouattara AK, Ouedraogo M (2018). Oncogenic human papillomavirus infection and genotypes characterization among sexually active women in Tenkodogo at Burkina Faso. West Africa Papillomavirus Res.

[135] Naud PS, Roteli-Martins CM, De Carvalho NS, Teixeira JC, de Borba PC, Sanchez N (2014). Sustained efficacy, immunogenicity, and safety of the HPV-16/18 AS04-adjuvanted vaccine: final analysis of a long-term follow-up study up to 9.4 years post-vaccination. Hum Vaccines Immunother.

[136] Huh WK, Joura EA, Giuliano AR, Iversen O-E, de Andrade RP, Ault KA (2017). Final efficacy, immunogenicity, and safety analyses of a nine-valent human papillomavirus vaccine in women aged 16-26 years: a randomised, double-blind trial. Lancet Lond Engl.

[137] Traore IMA, Zohoncon TM, Dembele A, Djigma FW, Obiri-Yeboah D, Traore G (2016). Molecular characterization of high-risk human papillomavirus in women in Bobo-Dioulasso. Burkina Faso BioMed Res Int.

[138] CMR O, Djigma FW, Bisseye C, Sagna T, Zeba M, Ouermi D (2011). Epidemiology, characterization of genotypes of human papillomavirus in a population of women in Ouagadougou. J Gynecol Obstet Biol Reprod (Paris).

[139] Ouédraogo C, Zohoncon TM, Traoré E, Ouattara S, Bado P, Ouedraogo C (2016). Distribution of high-risk human papillomavirus genotypes in precancerous cervical lesions in Ouagadougou, Burkina Faso. Clin Obstet Gynecol Reprod Med.

[140] Djigma FW, Ouédraogo C, Karou DS, Sagna T, Bisseye C, Zeba M (2011). Prevalence and genotype characterization of human papillomaviruses among HIV-seropositive in Ouagadougou. Burkina Faso Acta Trop.

[141] Zohoncon TM, Ouedraogo TC, Brun LVC, Obiri-Yeboah D, Djigma WF, Kabibou S (2016). Molecular epidemiology of high-risk human papillomavirus in high-grade cervical intraepithelial Neoplasia and in cervical Cancer in Parakou, Republic of Benin. Pak J Biol Sci PJBS.

[142] Piras F, Piga M, De Montis A, Zannou AR, Minerba L, Perra MT (2011). Prevalence of human papillomavirus infection in women in Benin. West Africa Virol J.

[143] Jaquet A, Horo A, Charbonneau V, Ekouevi DK, Roncin L, Toure B (2012). Cervical human papillomavirus and HIV infection in women of child-bearing age in Abidjan, Côte d’Ivoire, 2010. Br J Cancer.

[144] Ndiaye C, Alemany L, Ndiaye N, Kamaté B, Diop Y, Odida M (2012). Human papillomavirus distribution in invasive cervical carcinoma in sub-Saharan Africa: could HIV explain the differences?. Trop Med Int Health TM IH.

[145] Zohoncon TM, Bisseye C, Djigma FW, Yonli AT, Compaore TR, Sagna T (2013). Prevalence of HPV high-risk genotypes in three cohorts of women in Ouagadougou (Burkina Faso). Mediterr J Hematol Infect Dis.

[146] Roden RBS, Stern PL (2018). Opportunities and challenges for human papillomavirus vaccination in cancer. Nat Rev Cancer.

[147] Pastrana DV, Gambhira R, Buck CB, Pang Y-YS, Thompson CD, Culp TD (2005). Cross-neutralization of cutaneous and mucosal papillomavirus types with anti-sera to the amino terminus of L2. Virology.

[148] Alphs HH, Gambhira R, Karanam B, Roberts JN, Jagu S, Schiller JT (2008). Protection against heterologous human papillomavirus challenge by a synthetic lipopeptide vaccine containing a broadly cross-neutralizing epitope of L2. Proc Natl Acad Sci U S A.

[149] Schellenbacher C, Roden RBS, Kirnbauer R (2017). Developments in L2-based human papillomavirus (HPV) vaccines. Virus Res.

[150] Biemelt S, Sonnewald U, Galmbacher P, Willmitzer L, Müller M (2003). Production of human papillomavirus type 16 virus-like particles in transgenic plants. J Virol.

[151] Kohl T, Hitzeroth II, Stewart D, Varsani A, Govan VA, Christensen ND (2006). Plant-produced cottontail rabbit papillomavirus L1 protein protects against tumor challenge: a proof-of-concept study. Clin Vaccine Immunol.

[152] Hitzeroth II, Christensen ND, Rybicki EP, Kohl TO (2007). Expression of HPV-11 L1 protein in transgenic Arabidopsis thaliana and Nicotiana tabacum. BMC Biotechnol.

[153] Maclean J, Koekemoer M, Olivier AJ, Stewart D, Hitzeroth II, Rademacher T (2007). Optimization of human papillomavirus type 16 (HPV-16) L1 expression in plants: comparison of the suitability of different HPV-16 L1 gene variants and different cell-compartment localization. J Gen Virol.

[154] Matić S, Masenga V, Poli A, Rinaldi R, Milne RG, Vecchiati M (2012). Comparative analysis of recombinant human papillomavirus 8 L1 production in plants by a variety of expression systems and purification methods. Plant Biotechnol J.

[155] Varsani A, Williamson A-L, Stewart D, Rybicki EP (2006). Transient expression of human papillomavirus type 16 L1 protein in Nicotiana benthamiana using an infectious tobamovirus vector. Virus Res.

[156] Warzecha H, Mason HS, Lane C, Tryggvesson A, Rybicki E, Williamson A-L (2003). Oral immunogenicity of human papillomavirus-like particles expressed in potato. J Virol.

[157] What is Viral Hepatitis? | Division of Viral Hepatitis | CDC. 2019. https://www.cdc.gov/hepatitis/abc/index.htm. .

[158] Murphy DG, Sablon E, Chamberland J, Fournier E, Dandavino R, Tremblay CL (2015). Hepatitis C virus genotype 7, a new genotype originating from Central Africa. J Clin Microbiol.

[159] Ishii S, Koziel MJ (2008). Immune responses during acute and chronic infection with hepatitis C virus. Clin Immunol Orlando Fla.

[160] Lechner F, Gruener NH, Urbani S, Uggeri J, Santantonio T, Kammer AR (2000). CD8+ T lymphocyte responses are induced during acute hepatitis C virus infection but are not sustained. Eur J Immunol.

[161] Yerly D, Heckerman D, Allen TM, Chisholm JV, Faircloth K, Linde CH (2008). Increased cytotoxic T-lymphocyte epitope variant cross-recognition and functional avidity are associated with hepatitis C virus clearance. J Virol.

[162] Pniewski T, Kapusta J, Bociąg P, Wojciechowicz J, Kostrzak A, Gdula M (2011). Low-dose oral immunization with lyophilized tissue of herbicide-resistant lettuce expressing hepatitis B surface antigen for prototype plant-derived vaccine tablet formulation. J Appl Genet.

[163] Assih M, Ouattara AK, Diarra B, Yonli AT, Compaore TR, Obiri-Yeboah D (2018). Genetic diversity of hepatitis viruses in west-African countries from 1996 to 2018. World J Hepatol.

[164] Kramvis A, Kew MC (2007). Epidemiology of hepatitis B virus in Africa, its genotypes and clinical associations of genotypes. Hepatol Res Off J Jpn Soc Hepatol.

[165] Ayele S, Chataway J, Wield D (2006). Partnerships in African crop biotech. Nat Biotechnol.

[166] Cohen JI (2005). Poorer nations turn to publicly developed GM crops. Nat Biotechnol.

[167] Achour M, Younes BR, Kochbati L, Kahla S, Zeghal D, Maalej M, et al. Production of recombinant proteins GST L1, E6 and E7 tag HPV 16 for antibody detection of Tunisian cervical cancer patients. Afr J Biotechnol. 2009;8:369.

[168] Tombari W, Ghram A (2016). Production of a truncated recombinant HA1 for influenza a H9 subtype screening. Biol J Int Assoc Biol Stand.

[169] Thomson JA (2008). The role of biotechnology for agricultural sustainability in Africa. Philos Trans R Soc B Biol Sci..

[170] Halley-Stott RP, Tanzer F, Martin DP, Rybicki EP (2007). The complete nucleotide sequence of a mild strain of bean yellow dwarf virus. Arch Virol.

[171] Obembe OO (2010). The plant biotechnology flight: is Africa on board?. Afr J Biotechnol.

[172] Brugidou C, Holt C, Ngon A, Yassi M, Zhang S, Beachy R, Fauquet C (1995). Synthesis of an infectious full-length cDNA clone of rice yellow mottle virus andmutagenesis of the coat protein. Virology.

[173] Koala M, Traoré VSE, Sérémé D, Neya BJ, Brugidou C, Barro N (2017). Imperata yellow mottle virus: an emerging threat to maize, Sorghum and Pearl Millet in Burkina Faso. Agric Sci.

[174] Tiendrébéogo F, Lefeuvre P, Hoareau M, Traoré VSE, Barro N, Péréfarres F (2011). Molecular and biological characterization of pepper yellow vein Mali virus (PepYVMV) isolates associated with pepper yellow vein disease in Burkina Faso. Arch Virol.

[175] Tiendrebeogo Fidele, Traore V.S. Edgar, Barro Nicolas, Traore Alfred S., Konate Gnissa, Traore Oumar (2008). Characterization of Pepper yellow vein mali virus in Capsicum sp. in Burkina Faso. Plant Pathology Journal.

[176] Sérémé D, Lacombe S, Konaté M, Pinel-Galzi A, Traoré VSE, Hébrard E (2008). Biological and molecular characterization of a putative new sobemovirus infecting Imperata cylindrica and maize in Africa. Arch Virol.

[177] Secretariat of the Convention on Biological Diversity (2011). Nagoya protocol on access to genetic resources and the fair and equitable sharing of benefits arising from their utilization.

